# Seasonal plasticity of thermal tolerance indicates resilience to future climate in Australian damselflies

**DOI:** 10.1007/s00442-025-05745-w

**Published:** 2025-06-24

**Authors:** Md Tangigul Haque, Shatabdi Paul, Marie E. Herberstein, Md Kawsar Khan

**Affiliations:** 1 School of Natural Sciences, Macquarie University, North Ryde, 2109 Australia; 2 Department Biology, Chemistry and Pharmacy, Freie Universität Berlin, 14195 Berlin, Germany

**Keywords:** Climate change, Thermal tolerance, Insects, Seasonal change, Phenotypic plasticity

## Abstract

An animal’s response to climate warming is predominantly governed by its thermal tolerance. Seasonal temperature variation may indicate the boundaries of plasticity in insect thermal tolerance, which could predict the capacity to adapt to future climates. Here, we assess the changes in thermal breadth (the difference between the critical thermal maximum (CTmax) and critical thermal minimum (CTmin)) to estimate the thermal safety margin in *Ischnura heterosticta* and *Xanthagrion erythroneurum* damselflies across different seasons. For both species, CTmax and CTmin increased with monthly temperature, with a stronger increase of CTmin in summer. Overall, thermal breadth was broad in spring and autumn (around 41 degrees) but in summer we observed a large number of individuals with substantially narrower thermal breadth (down to 26–35 degrees). Our results establish considerable seasonal thermal plasticity in damselflies, which might provide a degree of resilience in future climates, yet during the most critical season (summer), heat spikes might push a substantial proportion of the population beyond their limits.

## Introduction

Anthropogenic climate change is contributing to the increase of temperature and temperature fluctuations across spatial scales (Sunday et al. 2014) and different seasons (Oliveira et al. 2021). These abrupt changes in temperature can affect individuals and populations directly, and also the way they interact with biotic and abiotic factors which ultimately impact biodiversity (Cadena et al. 2023) and ecosystems (Weiskopf et al. 2020). Populations capable of coping with these higher temperatures can persist in situ (Cancellario et al. 2022), or shift their range based on physiological thermal limits for survive and growth (Sheridan and Bickford 2011; Suhling and Suhling 2013; McCauley et al. 2015; Diamond 2018), development (Liefting et al. 2009), and reproduction (Zeh et al. 2014).

One of the major steps to understand population vulnerability is to determine the ability to withstand varying temperatures, which is known as thermal tolerance. Critical thermal maximum (CTmax) and critical thermal minimum (CTmin) represent the maximum and minimum temperature at which individuals can perform their activities without any additional physiological costs (Schulte et al. 2011; Dowd et al. 2015). CTmax and CTmin are used to quantify thermal tolerance—the breadth of temperatures within which individuals and populations can survive. When environmental temperatures are close to or exceed the limits of thermal breadth, metabolic efficiency and physiological performance decline and may result in death if prolonged (Dowd et al. 2015; Molnár et al. 2017).

Thermal acclimation, the shift in critical thermal limits following exposure to extreme but non-lethal temperatures, may buffer insect populations against the detrimental effects of climate change, especially in regions with high seasonal temperature variation (Guderley and St-Pierre 2002; Vinagre et al. 2016). For example, when *Drosophila melanogaster* was cold acclimated (both adult and larval stages), their tolerance to cold stress increased (Colinet and Hoffmann 2012). Another example showed that short-term heat acclimatization of rice leaf folder larvae *Cnaphalocrocis medinalis* for several generations can improve the heat tolerance of their offspring (Gu et al. 2019). However, while insects appear to express consistent thermal plasticity, there is limited evidence that this facilitates survival at extreme temperatures (Weaving et al. 2022).

Daily and seasonal temperature fluctuations may facilitate thermal plasticity in insect populations and provide indications of how they might respond to the predicted temperature increases over the next decades (Leclair et al. 2020). During summer, increasing temperatures may lead to high CTmax (see review Roeder et al. 2021), thereby acclimatizing individuals to temperature spikes, but at the same time, overall thermal breadth might decrease, because the CTmin also increases. Populations with a narrower thermal breadth are at greatest risk of extinctions than populations with broader thermal breadth, due to a reduced physiological performance outside this narrow thermal breadth (Pörtner and Farrell 2008).

A useful indicator of population thermal vulnerability is the thermal safety margin—the difference between critical thermal limits (CTmax and CTmin respectively) and the maximum temperature of the warmest month and the minimum temperature of the coldest month (Sunday et al. 2014). While there are many studies that quantify how latitudinal and elevational changes in temperature affect thermal limits (see Weaving et al. 2022 for a meta-analysis), there is relatively little information on how seasonal variation in temperature impacts thermal limits.

Dragonflies and damselflies (Odonata) are freshwater insects that are considered as barometers for climate change (Hassall 2015). Variation in environmental temperatures impact odonates in several ways, including their life history traits and foraging activity (De Block and Stoks 2003; Paul et al. 2024), phenology (Hassall et al. 2007), distribution (Hassall and Thompson 2010), developmental rate (Hassall and Thompson 2008), and thermal limits (Castillo-Pérez et al. 2022). For example, survivability of the damselfly *Ischnura elegans* in cold weather conditions increased when treated with short-term extreme low temperature (simulating a cold wave), however, an adverse effect was observed during long-term exposure to both high and low extreme temperatures (Smith and Lancaster 2020).

The objective of our study is to understand two key aspects of the thermal biology of damselflies: (i) the impact of seasonal variation on CTmax, CTmin, and thermal breadth; and (ii) the thermal safety margin to predict the vulnerability of damselfly populations under future temperature scenarios. We hypothesize that CTmax, CTmin, and thermal breadth of damselflies varies throughout the season and predict that their thermal safety margin is reduced in seasons with higher temperatures (Ma et al. 2021).

## Materials and methods

### Study species

*Ischnura heterosticta* and *Xanthagrion erythroneurum* are species of non-territorial damselfly (Coenagrionidae: Zygoptera: Odonata), commonly found on ponds, lakes, marshes, and lagoons throughout Australia (Theischinger and Hawking 2006). Adult *Ischnura heterosticta* males have a bright blue thorax and blue stripes on abdominal segments eight and nine (Huang et al. 2012), whereas adult *Xanthagrion erythroneurum* males have a red thorax, red abdominal stripes on segment one and two, and blue abdominal stripes on segments eight and nine (Theischinger and Hawking 2016; Khan and Herberstein 2019). *Ischnura heterosticta* females undergo ontogenetic colour change, having a blue thorax and blue stripes on abdominal segments eight and nine on pre-reproductive andromorph females, whereas adult heteromorph females have a grey thorax and abdomen (Huang et al. 2012). *Xanthagrion erythroneurum* males and females also exhibit ontogenetic colour changes from yellow to red (Khan and Herberstein 2020, 2021). The flight activity period of *Ischnura heterosticta* is usually between October and March (Huang et al. 2012) whereas *Xanthagrion erythroneurum*’s flight activity spans from September to April (Khan and Herberstein 2020).

### Study sites and specimen collection

We collected damselflies on a sunny and partially sunny days from a pond located at Macquarie University (33.772 S, 151.114 E) and Student Village (33.770 S, 151.106 E) in North Ryde, Australia, the lands of the Wallumattagal Clan of the Dharug Nation. Two sites were approximately 500 m apart. We captured both species every fortnight using an insect sweep net (dimensions: 1260 mm handle, 456 mm diameter hoop, 456 mm diameter polyester bag) between 09:00 and 13:00 h from December 2022 to December 2023. We collected a total of 1536 damselflies (80 individuals; 40 for *Ischnura heterosticta* and 40 for *Xanthagrion erythroneurum f*rom each site in each sampling day) based on their availability. We transported damselflies from field sites to Macquarie University Behaviour Ecology lab using mesh travel containers (Diameter: 14 cm, Height: 23 cm (expanded), 1.5 cm (packed). No permission was required to conduct this fieldwork as the species were not protected in New South Wales and sampling sites were not part of protected areas.

**Fig. 1:**
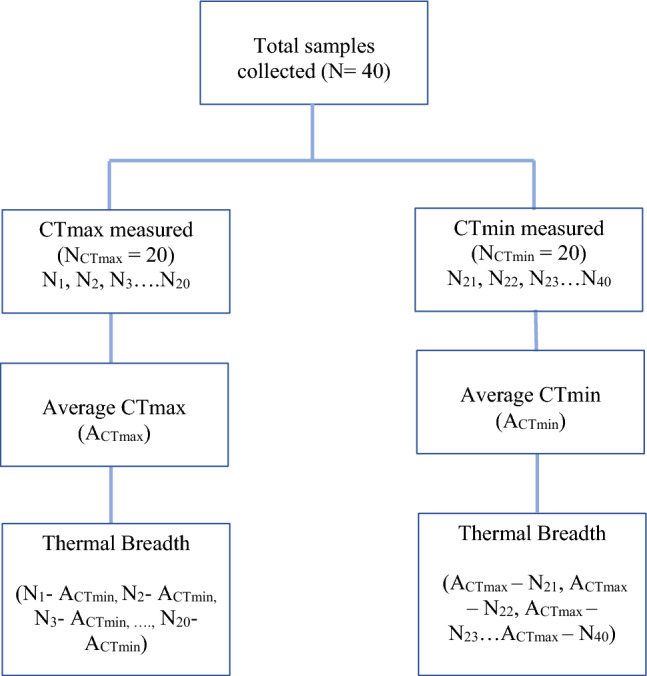
This figure represents the procedure used to calculate thermal breadth in our study

Because we could not measure CTmax and CTmin in the same individual we randomly allocated 20 individuals of each species to the ‘CTmax group’ and the other 20 to the ‘CTmin group’. We then calculated the average CTmax and CTmin (respectively) from these groups for calculating thermal breadth (Fig. 1).

### CTmax, CTmin, thermal breadth, and thermal safety margin measurement

We measured damselfly CTmax using dynamic ramping method. We placed active and undamaged damselflies (e.g. no wings broken) into a 15 ml centrifuge tube (Sarstedt AG & Co. KG, Nümbrecht, Germany), and submerged the tubes in a water bath (model: MyBath™ mini water bath, Benchmark Scientific B2000-4-T5; accuracy: ± 0.5 °C). We ensured the accuracy of the water bath temperature by using an additional external thermometer (model-TP300, ThermoPro). We kept damselflies in the water bath for 15 min at 25 °C, and then increased the water bath temperature by 1 °C which took on average (2.6 ± 0.25 SE) minutes. When the temperature increased one degree, we kept damselflies at that new temperature for 3 min to acclimatize to the new temperature, and then checked damselfly activity. We continued this process until the damselfly was knocked down, and recorded the knockdown temperature as CTmax.

For CTmin, we performed the similar process on the second set of 20 individuals per species, except decreasing the water bath temperature by 1 °C. The damselflies were placed in a tube and acclimatized in the water bath at 25 °C for 15 min. Then we moved the tubes into a refrigerator at ~ − 4 °C, which took around 150 min. We checked the status of each damselfly at every 1 min after the water reached 15 °C (as from our preliminary experiment we know that CTmin is usually less than 11 °C in our study species). We continued this process until a damselfly was knocked down and recorded the knockdown temperature as CTmin. Finally, we stored the damselfly at − 30° C in 95% ethanol for morphological measurement.

For calculating the thermal breadth, we subtracted each individual CTmax measurement from the ‘CTmax group’ from the average CTmin generated by the ‘CTmin group’ and conversely, we subtracted each individual CTmin measurement (CTmin group) from the average CTmax (CTmax group).

1 $${\text{Thermal Breadth }}^{\prime } {\text{CTmax group}}^{\prime } {\mkern 1mu} = {\mkern 1mu} {\text{CTmax}}_{{({\mathbf{individual}})}} {\text{ - CTmin}}_{{({\mathbf{average}}{\kern 1pt} ^{\prime } {\mathbf{CTmin}}{\kern 1pt} {\mathbf{group}}^{\prime } )}}$$

2 $${\text{Thermal Breadth }}^{\prime } {\text{CTmin group}}^{\prime } {\mkern 1mu} = {\mkern 1mu} {\text{CTmax}}_{{({\mathbf{average}}{\kern 1pt} {\mathbf{CTmax}}{\kern 1pt} {\mathbf{group}})}} - {\text{CTmin}}_{{({\mathbf{individual}})}}$$

Finally, we measured the future thermal safety margin for these damselflies using two approaches- a) for CTmax- subtracting the maximum predicted temperature of the warmest month from individual CTmax (CTmax – BIO5), and b) for CTmin- subtracting CTmin from the minimum predicted temperature of coldest month (BIO6—CTmin).

We used a water bath for measuring CTmax and CTmin of damselflies and our experimental design did not involve any irradiation. Therefore, absorption or reflection of light did not influence CTmax and CTmin.

### Body mass measurement

We measured the body mass of the specimens and stored in 95% ethanol in a -30 freezer. We first placed the damselfly on an absorbent paper for two minutes to evaporate the ethanol (Khan 2020). Next, we measured body mass of each damselfly using a Mettler Toledo analytical balance (model ML204T/00, accuracy 0.0001 g).

### Climatic variables

We extracted the daily average temperature of the day of specimen collection and monthly average temperature for our sampling sites from the nearest weather station from Australian Government Bureau of Meteorology (BOM) (http://www.bom.gov.au). Government Bureau of Meteorology uses same weather station for determining climatic data for both sites, therefore, climatic variables were same for both sites. Next, we downloaded two bioclimatic variables (maximum temperature of warmest month (BIO5) and minimum temperature of coldest month (BIO6)) data for predicting future thermal safety margins for the years 2080–2100 from the WorldClim database version 2.1 (https://www.worldclim.org) with a spatial resolution of 30 s (Fick and Hijmans 2017). We used the global climate model (ACCESS-CM2) and Shared Socio-economic Pathways (ssp585) to predict future climate data. We chose this model because it is run by Commonwealth Scientific and Industrial Research Organisation (CSIRO), Australia and it is an up-to-date model for simulating climate up to 2100 for a range of future Shared Socio-economic Pathways (SSPs) (Bi et al. 2020). Next, we used the R package “raster” to extract climate data from the database (Hijmans and van Etten 2012).

### Statistical analyses

We conducted all of our analyses using R version 4.1.2 (R Core Team 2021). We quantified the differences of thermal breadth, CTmax, and CTmin across different seasons using DurgaDiff function of R package “Durga” (Khan and McLean 2024). We applied generalized linear mixed models using template model builder (glmmTMB) to assess the impact of climatic variable (daily average temperature), body weight, and sex on CTmax and CTmin. We fitted models using CTmax or CTmin as a response variable and season, daily average temperature, body weight, and sex as a fixed factor while month as a random factor. Then, we checked the goodness-of-fits of models using the R package “DHARMa” (Hartig 2020). Finally, we projected the vulnerability of damselflies to rising temperature by assessing their thermal safety margins across different seasons. All data are presented as mean ± SE.

## Results

We captured a total of 953 (male = 509 and female = 444) *Ischnura heterosticta* and 583 (male = 467 and female = 116) *Xanthagrion erythroneurum* damselflies from the two sites. For *Ischnura heterosticta*, the average body weight of females (40.09 ± 0.38 mg) was greater than that of males (32.43 ± 0.25 mg). Similarly, for *Xanthagrion erythroneurum*, the average body weight of females (40.57 ± 0.74 mg) was greater than that of males (29.71 ± 0.21 mg). CTmax, CTmin and thermal breadth varies across seasons for both species (Table 1). The trend of thermal tolerance limit data was similar for sites, and climatic data such as average daily temperature and monthly temperature was same for both sites. Therefore, for all analyses, data from both sites were pooled and analysed together.

**Table 1:** CTmax, CTmin, and thermal breadth of *Ischunra heterosticta* and *Xanthagrion erythroneurum* across different seasons

Species	Season	CTmax (± SE) °C	CTmin (± SE) °C	Thermal breadth (± SE) °C
*Ischunra heterosticta*	Spring	43.4 ± 0.04	2.87 ± 0.06	40.6 ± 0.05
	Summer	43.01 ± 0.07	3.07 ± 0.09	39.9 ± 0.06
	Autumn	43.3 ± 0.09	3.2 ± 0.13	40.0 ± 0.08
*Xanthagrion erythroneurum*	Spring	43.7 ± 0.06	3.6 ± 0.06	40.1 ± 0.03
	Summer	43.0 ± 0.12	4.2 ± 0.18	38.8 ± 0.11
	Autumn	43.7 ± 0.07	4.1 ± 0.18	39.5 ± 0.11

### CTmax variation across seasons

**Fig. 2:**
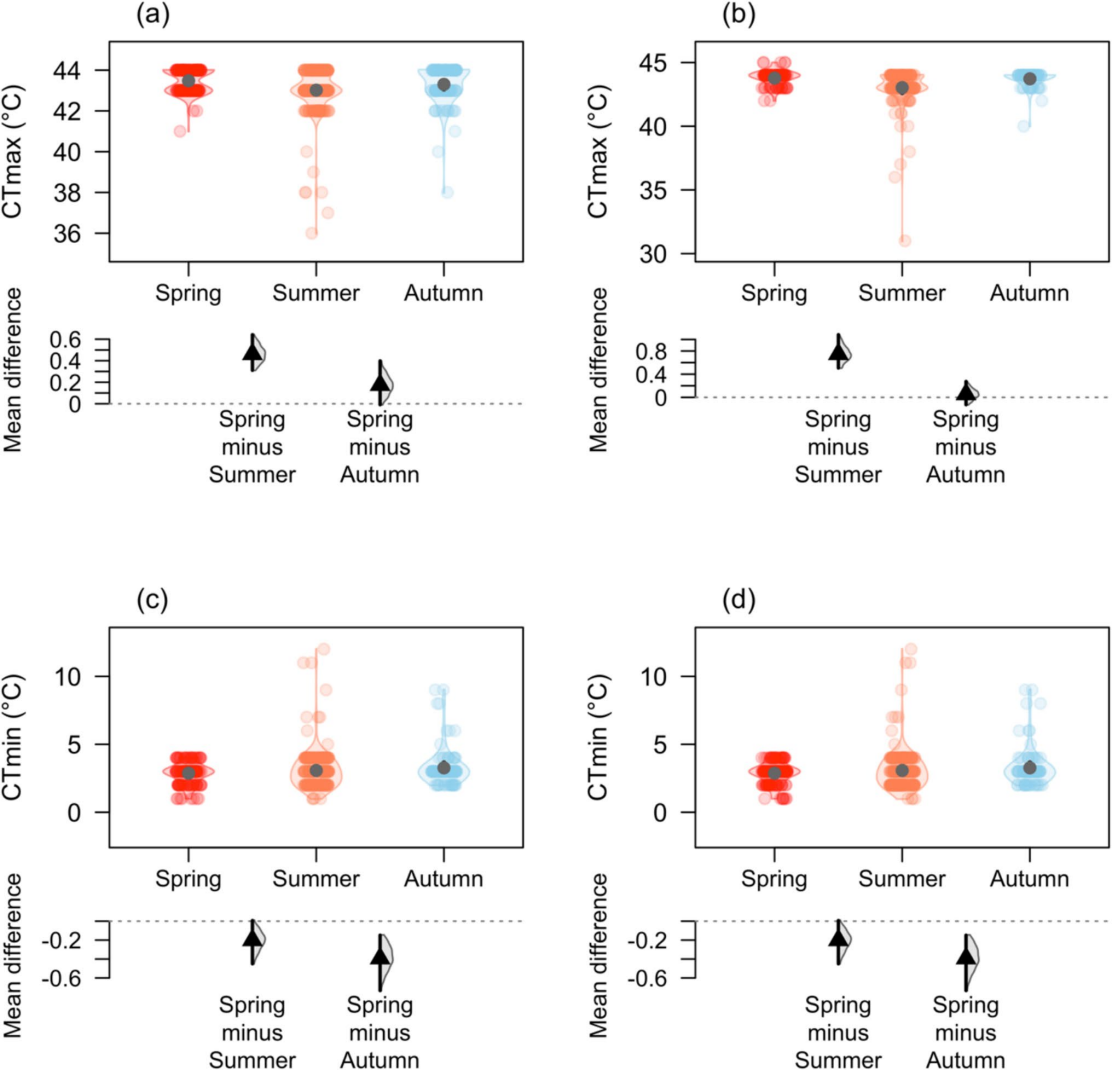
DurgaPlot (Khan & McLean 2024) showing CTmax of **a** *Ischunra heterosticta* and **b** *Xanthagrion erythroneurum* for spring, summer, and autumn. CTmin of **c** *Ischunra heterosticta* and **d** *Xanthagrion erythroneurum* for spring, summer, and autumn. Small circles, irrespective to colours, represent different CTmax or CTmin in different seasons and the black dot denotes the mean of CTmax or CTmin. The bottom panels represent mean differences in CTmax or CTmin across different seasons

**Fig. 3:**
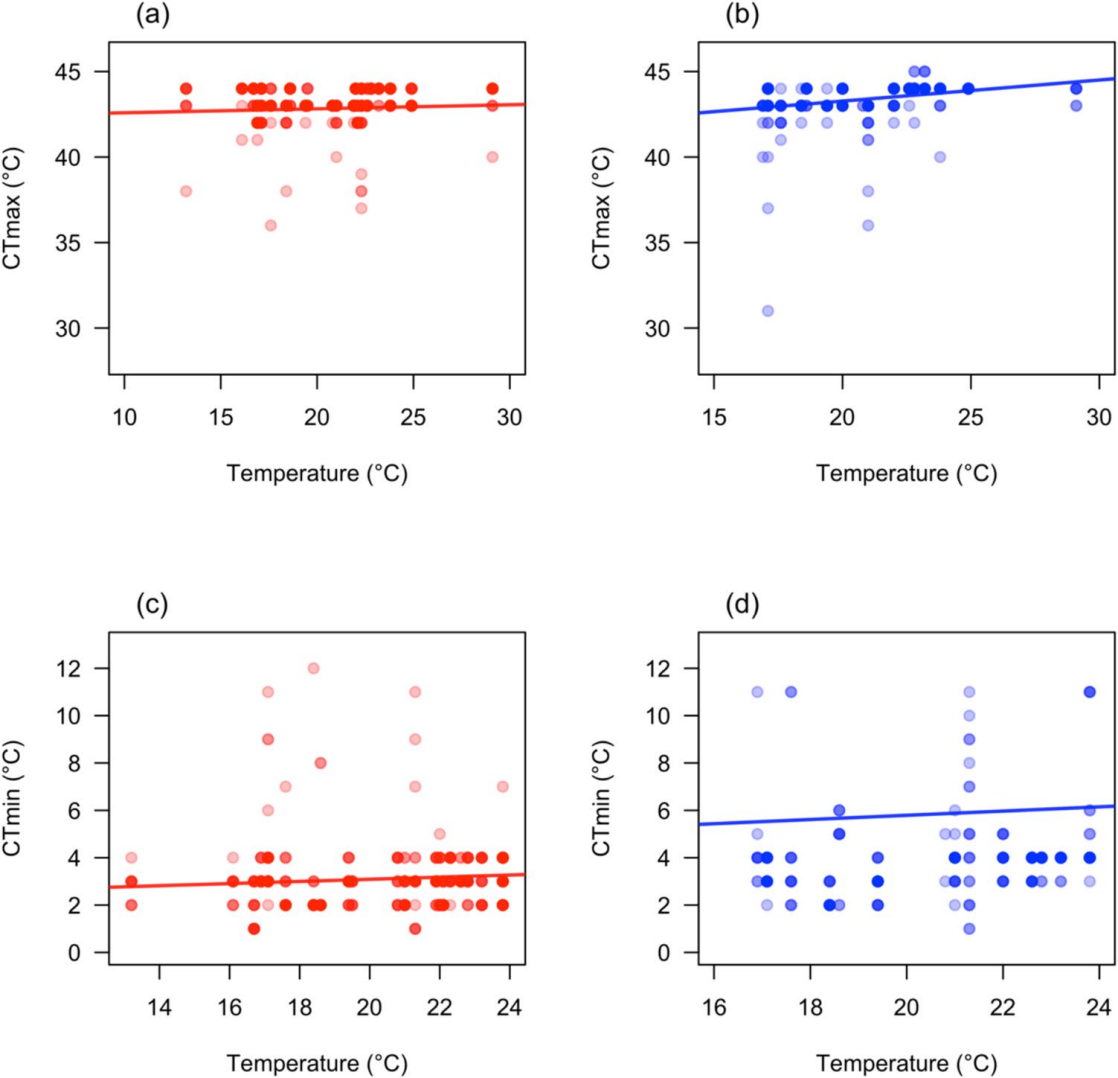
CTmax of **a** *Ischnura heterosticta* and **b** *Xanthagrion erythroneurum* increases with increasing daily average temperature. CTmin of (**c** *Ischnura heterosticta* and **d** *Xanthagrion erythroneurum* increasing with daily average temperature. Small circles represent individuals CTmax (**a** and **b**) and CTmin (**c** and **d**). Dark circle denotes many individuals have the same CTmax or CTmin and light circle represents individuals that have same CTmax or CTmin

The average CTmax of *Ischnura heterosticta* was higher in spring (43.4 ± 0.04 °C) compared to summer (43.0 ± 0.07 °C) and autumn (43.3 ± 0.09 °C; Fig. 2a). CTmax increased with increasing daily average temperature (glmmTMB: estimate: 0.0431 ± 0.015, Z = 2.72, P = 0.01, Fig. 3a) irrespective of the season, however, CTmax was not impacted by body weight (glmmTMB: estimate: − 0.0004 ± 0.006, Z = − 0.06, P = 0.95), or sex (glmmTMB: estimate: 0.0312 ± 0.098, Z = 0.32, P = 0.75).

Similarly, the average CTmax of *Xanthagrion erythroneurum* was highest in spring (43.7 ± 0.06 °C) compared to summer (43.0 ± 0.12 °C) and autumn (43.7 ± 0.07 °C; Fig. 2b). In addition, we found that CTmax increased with increasing daily average temperature (glmmTMB: estimate: 0.137 ± 0.03, Z = 4.61, P = 0.001, Fig. 3b) throughout the year, however, body weight (glmmTMB: estimate: 0.016 ± 0.012, Z = 1.31, P = 0.19) and sex (glmmTMB: estimate: -0.067 ± 0.214, Z = -0.32, P = 0.75) had no impact on CTmax.

### CTmin variation across seasons

We found the lowest CTmin of *Ischnura heterosticta* in spring (2.8 ± 0.06 °C) compared to summer (3.0 ± 0.09 °C) and autumn (3.2 ± 0.13 °C; Fig. 2c). In addition, we found CTmin increased with increasing daily average temperature (glmmTMB: estimate: 0.053 ± 0.022, Z = 2.39, P = 0.02, Fig. 3c), and males had a higher CTmin than females (glmmTMB: estimate: 0.357 ± 0.136, Z = 2.62, P = 0.01). However, body weight (glmmTMB: estimate: -0.005 ± 0.009, Z = − 0.59, P = 0.55) had no impact on *Ischnura heterosticta*’s CTmin.

Similarly, we found lowest CTmin of *Xanthagrion erythroneurum* in spring (3.6 ± 0.06 °C) compared to autumn (4.1 ± 0.18 °C) and summer (4.2 ± 0.18 °C; Fig. 2d). CTmin increased with increasing daily average temperature (glmmTMB: estimate: 0.089 ± 0.033, Z = 2.65, P = 0.01, Fig. 3d), and individual with lower body weight had higher CTmin (glmmTMB: estimate: − 0.044 ± 0.02, Z = − 2.12, P = 0.03). But *Xanthagrion erythroneurum* CTmin did not vary between sexes (glmmTMB: estimate: − 0.493 ± 0.34, Z = − 1.45, P = 0.14).

### Thermal breadth variation across seasons

**Fig. 4:**
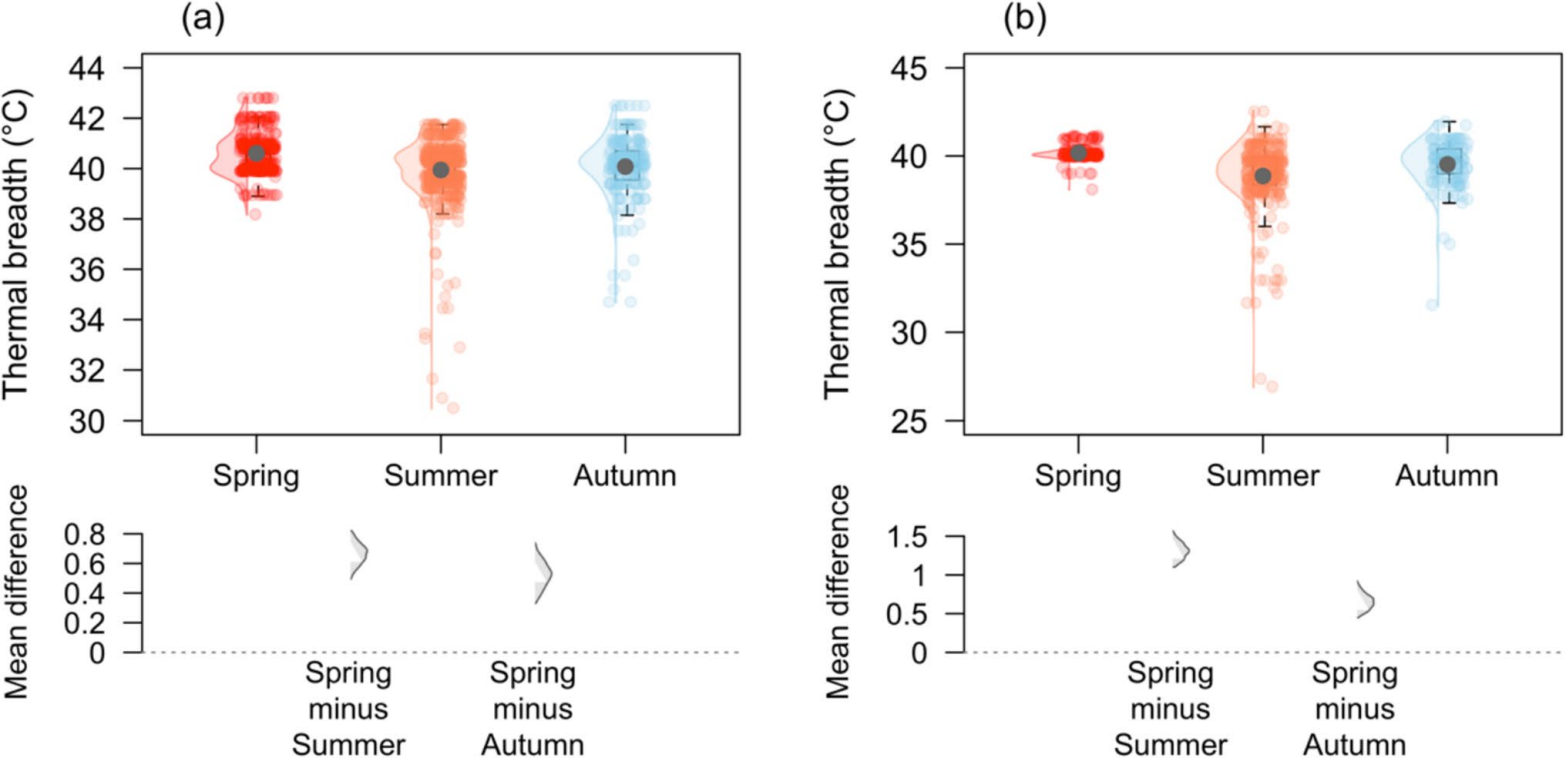
*Ischunra heterosticta* (**a**) and *Xanthagrion erythroneurum* (**b**) showed narrow thermal breadth during summer and wide thermal breadth in spring. Small circles, irrespective to colours, represent different thermal breadth at different seasons and black dot denotes the mean of thermal breadth. The lower panels represent mean differences in thermal breadth across different seasons. The box plots display the group median and the 75th and 25th percentiles. The whiskers extend to the minimum and maximum values

We found *Ischnura heterosticta* had the widest thermal breadth in spring (40.6 ± 0.05 °C) which narrowed in autumn (40.0 ± 0.08 °C) and summer (39.9 ± 0.06 °C) (Fig. 4a). Similarly, we found *Xanthagrion erythroneurum* had a wide thermal breadth in spring (40.1 ± 0.03 °C) which narrowed in autumn (39.5 ± 0.11 °C) and summer (38.8 ± 0.11 °C; Fig. 4b).

### Vulnerability of damselfly due to future temperature fluctuations

**Fig. 5:**
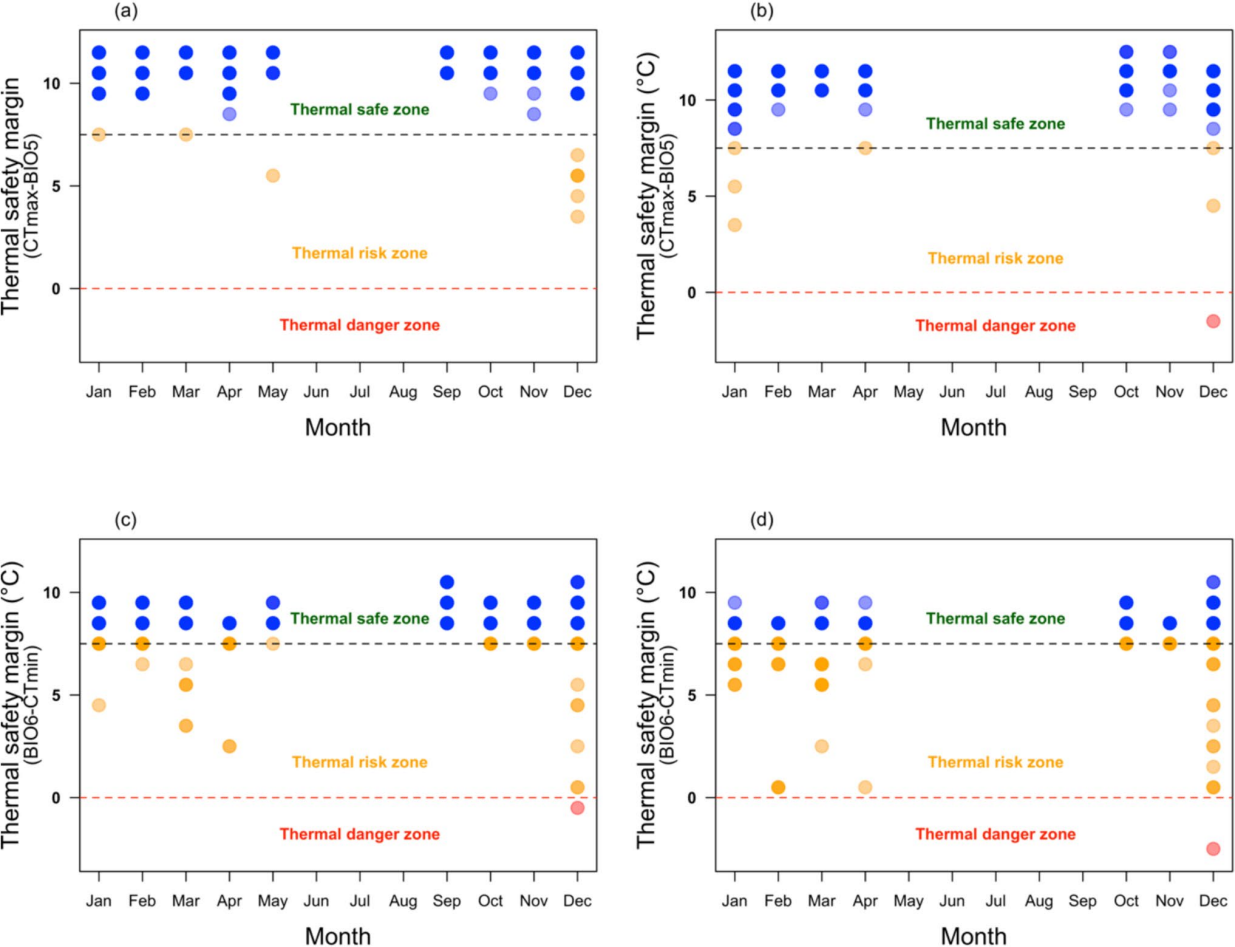
Thermal safety margins for *Ischunra heterosticta* under BIO5 (**a**) and BIO6 (**c**) predictions and thermal safety margins for *Xanthagrion erythroneurum* under BIO5 (**b**) and BIO6 (**d**) predictions. Zone under the horizontal red dotted line is the thermal danger zone, between the red dotted and black dotted line is the thermal risk zone, and above the horizonal black dotted line is the thermal safe zone. Red circles denote individuals that are in the thermal danger zone, orange circles denote individuals that are in the thermal risk zone, and blue circles denotes individuals that are in the thermal safe zone

Our projection data showed that thermal safety margins will be narrower and, in some cases, even cross into the thermal danger zone in summer, making both species *Ischunra heterosticta* (Fig. 5a, c) and *Xanthagrion erythroneurum* (Fig. 5b, d) vulnerable to future climate change.

## Discussion

In our study, we quantified the thermal tolerance (CTmax and CTmin) of *Ischunra heterosticta* and *Xanthagrion erythroneurum* damselflies to estimate the thermal safety margin (CTmax minus maximum temperature of warmest month; minimum temperature of the coldest month minus CTmin) under future temperature scenarios. Additionally, we measured population-specific thermal breadth (CTmax minus CTmin) to determine the vulnerability of damselflies under current seasonal temperature fluctuations. The thermal safety margin for both species was lowest in summer. Both species had the broadest thermal breadth in spring and the narrowest in summer. CTmax and CTmin increased with increasing temperature regardless of seasons, sex or body mass.

While previous work on Australian damselflies (Haque et al. 2025) has found higher CTmax in populations with higher local temperatures, our observations across the season in a single population has revealed the opposite pattern: the highest CTmax was in spring and autumn, and the lowest in summer. Some species follow this counter-intuitive seasonal pattern (mosquito (Oliveira et al. 2021), lizard (Hodgson and Schwanz 2019) and skink (Llewelyn et al. 2017), while others had a higher CTmax in summer- ants (Bujan et al. 2020) and aquatic insects (Houghton and Shoup 2014). The lack of alignment between cross and within population responses to high temperature may reflect different adaptive processes: long term evolutionary responses to population specific climate and short-term plastic responses to seasonal fluctuations. Another reason is that populations in warmer climates may exhibit higher CTmax due to consistent heat exposure throughout their lifespan. This may select for individuals with higher CTmax, enabling them to function and survive under extreme conditions (Castillo-Pérez et al. 2022; Haque et al. 2025). In contrast, CTmax can vary seasonally within a single population- a lower CTmax in summer may be associated with an adaptive trade-off, such as energy conservation during excessive heat peaks and focusing on other activities e.g., growth and reproduction. Finally, the magnitude of warming including the intensity and frequency of extremes is higher in spring and autumn than in summer (Alexander et al. 2006; IPCC 2014) which may be responsible for higher CTmax in spring and autumn than summer in our study.

Focusing on the responses to seasonal changes in temperature, this might be the result of (1) behavioural changes whereby individuals avoid excess heat by minimizing exposure to high temperature in summer, leading to less acclimation or relaxed selection for high thermal tolerance (Oliveira et al. 2021); (ii) high CTmax in cooler environments may offer a selective advantage as it speeds-up the physiological processes (May 1998; Llewelyn et al. 2017) allowing individuals to remain active for longer periods; and (iii) high CTmax in cooler seasons may enable individuals to exploit warmer microhabitats (such as sunlit areas and near surface areas or littoral zone) for foraging, mating or avoiding predators. Unlike CTmax, CTmin varied predictably across seasons, being higher in summer and lower in autumn and spring (see also Sanabria et al. 2013; Sharma et al. 2015; Oliveira et al. 2021).

Thermal breadth decreased from spring to summer and then increased from summer to autumn, which is consistent with previous studies that found a wider thermal breadth during colder seasons and a narrower thermal breadth during warmer seasons (Oliveira et al. 2021; Clifton and Refsnider 2022). This seasonal pattern also extends to broader spatial scales, with tropical species having a narrower thermal breadth than temperate species (Shah et al. 2017; Dewenter et al. 2024). In spring, populations experience more variable temperatures due to the transition from colder to warmer conditions, which are likely to be mitigated by a broad thermal breadth in spring. Whereas the narrow thermal breadth in summer (mostly due to a higher CTmin) is likely a response to the relatively constant but higher summer temperatures, because the range of temperatures in summer is likely to be more predictable than in the other seasons (Fusi et al. 2024). For example, a slightly wider thermal breadth in spring and autumn may allow damselflies to remain active in a fluctuating thermal environment- cool mornings and warm afternoons. By contrast, damselflies emerging in summer may not need to tolerate low temperature in summer and thus express a more optimized or narrower thermal breadth with higher CTmin. An additional possible mechanism to explain within population seasonal shifts in thermal tolerance is the selection of seasonal polymorphisms (associated with dramatic shifts in allele frequencies) in response to temperature fluctuations (Bergland et al. 2014).

We observed relatively low variation in the thermal breadth of damselflies across seasons, possibly because we sampled damselflies from adjacent waterbodies with similar microhabitats which limits site-specific variation in thermal breadth. Nonetheless, even a subtle difference in thermal breadth may provide important ecological and selective advantages for damselflies. A recent meta-analysis on 138 ectothermic species showed that changes in 1 °C of developmental temperature increased species thermal tolerance by 0.13 °C (Pottier et al. 2022). Hence, a minor shift in thermal breadth can significantly influence survival rate, foraging activity, mating success rate, and daily activity patterns.

Our projections indicate that populations will have a lower thermal safety margin during summer than autumn and spring under future climate scenarios. Hence, damselflies (and possibly other insects) will be more vulnerable in the summer months due to higher fluctuations in temperatures (e.g., heat waves). Our findings support previous projections that also found narrower thermal safety margins under higher temperatures (Leclair et al. 2020; van der Walt et al. 2021). This may particularly affect populations in warmer areas (tropics) as they have a lower thermal safety margin than populations inhabiting cooler temperate regions (Deutsch et al. 2008; Morley et al. 2019; Haque et al. 2025). The seasonal changes in the thermal safety margin may be related to seasonal changes in CTmax which may be approached or even exceeded by the maximum temperatures during summer and autumn. With the predicted increase in the frequency of heat waves and average global temperature (Perkins-Kirkpatrick and Lewis 2020; Tripathy et al. 2023) a seasonal decline of populations is likely. Further studies focusing on the interactions with other factors such as life stages (Kingsolver et al. 2011; Kingsolver and Buckley 2020), different habitats (Gunderson and Stillman 2015), and fertility (Walsh et al. 2021; van Heerwaarden and Sgrò 2021) with thermal tolerance, and might help us to refined population vulnerability.

Insects use a variety of physiological mechanisms to cope with varying temperatures (Colinet et al. 2015). They can produce molecular chaperons or specific molecule to prevent protein denaturation or cell inactivation under extreme temperatures (Ma et al. 2021). For example, *Apis melifera jemenetica* expresses heat shock proteins (e.g., hsp10, hsp28, hsp70ab, hsp83 and hsp90) in the tropics in response to very high temperatures and these are thought to enhance survival and fitness (Alghamdi and Alattal 2023). Hormones also plays an important role in thermal adaptation by modulating physiology, development and behaviour (Emerson et al. 2009). For example, juvenile hormone and 20-hydroxy-ecdysone (20E) hormone increases the level of the enzyme alkaline phosphatase in insects which influence thermal tolerance (Rauschenbach et al. 2007). Finally, changes in insect enzymes activity levels according to seasons can increase thermal tolerance under extreme temperatures ( for review see Clark and Worland 2008). However, the exact mechanisms behind these physiological responses are still poorly understood. Alternatively, rather than adjusting physiological mechanisms with temperature extremes insects may adapt to a changing climate via phenological shifts than physiological adaptations (Gotthard et al. 2025).

In summary, our study suggests that damselflies might face more challenging conditions with seasonal extremes in summer compared to cooler seasons due to a narrower thermal breadth and a lower thermal safety margin during the hottest months of the year. The higher frequency of hot summers, heat waves and an increase of winter temperatures in the near future is likely to change seasonal abundance and contribute to the seasonal decline of populations in summer. Life-history evolution (including phenological shifts), behaviour (e.g. dispersal) and physiological adaptations (e.g. diapause, upregulation of enzymes and proteins) may mitigate some adverse effects, but these mechanisms may not fully eliminate the risk of local extinctions under extreme temperatures.

## Data Availability

The data that support the findings of this study is deposited in Figshare and can be accessed via following link: https://figshare.com/s/e8e742356e6692ef7010

## Code availability

All codes used to analyse data of this study is deposited in Figshare and can be accessed via following link: https://figshare.com/s/e8e742356e6692ef7010
